# The Role of Astrocytic Aquaporin-4 in Synaptic Plasticity and Learning and Memory

**DOI:** 10.3389/fnint.2016.00008

**Published:** 2016-02-24

**Authors:** Jenny I. Szu, Devin K. Binder

**Affiliations:** Center for Glial-Neuronal Interactions, Division of Biomedical Sciences, School of Medicine, University of California, Riverside, RiversideCA, USA

**Keywords:** aquaporin-4, synaptic plasticity, long term potentiation, long term depression, learning, memory

## Abstract

Aquaporin-4 (AQP4) is the predominant water channel expressed by astrocytes in the central nervous system (CNS). AQP4 is widely expressed throughout the brain, especially at the blood-brain barrier where AQP4 is highly polarized to astrocytic foot processes in contact with blood vessels. The bidirectional water transport function of AQP4 suggests its role in cerebral water balance in the CNS. The regulation of AQP4 has been extensively investigated in various neuropathological conditions such as cerebral edema, epilepsy, and ischemia, however, the functional role of AQP4 in synaptic plasticity, learning, and memory is only beginning to be elucidated. In this review, we explore the current literature on AQP4 and its influence on long term potentiation (LTP) and long term depression (LTD) in the hippocampus as well as the potential relationship between AQP4 and in learning and memory. We begin by discussing recent *in vitro* and *in vivo* studies using AQP4-null and wild-type mice, in particular, the impairment of LTP and LTD observed in the hippocampus. Early evidence using AQP4-null mice have suggested that impaired LTP and LTD is brain-derived neurotrophic factor dependent. Others have indicated a possible link between defective LTP and the downregulation of glutamate transporter-1 which is rescued by chronic treatment of β-lactam antibiotic ceftriaxone. Furthermore, behavioral studies may shed some light into the functional role of AQP4 in learning and memory. AQP4-null mice performances utilizing Morris water maze, object placement tests, and contextual fear conditioning proposed a specific role of AQP4 in memory consolidation. All together, these studies highlight the potential influence AQP4 may have on long term synaptic plasticity and memory.

## Introduction

Astrocytes were previously thought to act only as support cells, however, over the years, increasing amount of evidence have implicated more dynamic functions of these glial cells (Volterra and Meldolesi, 2005). It is now well known that astrocytes play various vital roles in the central nervous system (CNS) such as providing metabolic and structural support (Barker and Ullian, 2010; Scharfman and Binder, 2013) and regulating the blood-brain barrier (BBB; Abbott et al., 2006). In addition to these roles, multiple studies have also shown that astrocytes can impact synaptic plasticity. Modulation of synapses by astrocytes can be observed by their structural organization. Astrocytic processes contact multiple neuronal membranes and form the tripartite synapse where the astrocytic foot process envelopes the pre and postsynaptic terminal (Halassa et al., 2007, 2009). The intimate contact between astrocytes and neurons allow astrocytes to directly influence the strength of the synapse (Barker and Ullian, 2010). For example, astrocytes can secrete factors that can directly influence the formation of synapses (Barker and Ullian, 2010; Scharfman and Binder, 2013). Glia-derived secreted factors that promote synaptogenesis include cholesterol coupled to apolipoprotein-E (Mauch et al., 2001) and thrombospondins (Christopherson et al., 2005) whereas inhibitory synapses are influenced by astrocyte conditioned media by increasing vesicular GABA transporter and GABA_A_ receptor through TrkB signaling (Elmariah et al., 2005). One of the hallmarks of synaptic plasticity involves the trafficking of AMPA receptors (AMPAR) to the synaptic sites (Malinow and Malenka, 2002; Malinow, 2015). Astrocyte-released cytokine tumor necrosis factor-α (TNFα) is involved in increasing surface expression of AMPAR (Beattie et al., 2002). Furthermore, TNFα has been shown to be involved in homeostatic synaptic scaling where neurons adjust the strength of their synapses to maintain its electrical output (Stellwagen and Malenka, 2006; Achour and Pascual, 2010). Other factors, such as glutamate, ATP, and D-serine, are also released by astrocytes. Termed ‘gliotransmitters,’ these molecules can regulate neuronal and synaptic activity (Perea et al., 2009; Achour and Pascual, 2010; Barker and Ullian, 2010; Parpura and Zorec, 2010).

Taken together, these roles suggest that astrocytes may be a key player in synaptic plasticity by modulating neuronal function. Moreover, studies have proposed that long lasting changes in synaptic plasticity underlies the basis of learning and memory. For example, long term potentiation (LTP) has been linked to acquisition and learned behaviors (Lu et al., 2008). Therefore, while the role of astrocytes in cellular communication and synapse regulation may be well established, the role of astrocyte-specific proteins, specifically aquaporin-4 (AQP4), in synaptic plasticity and learning and memory remains unclear. In this review we explore current literature on AQP4 and its impact on LTP and long term depression (LTD) as well as the potential relationship between AQP4 and in cognitive functions.

## Aquaporin-4

The aquaporins (AQP) are a family of small, hydrophobic, membrane-spanning proteins involved in fluid transport (∼30 kDA/monomer; Verkman and Mitra, 2000; Verkman, 2005, 2008, 2011) Aquaporin-4 (AQP4) is one of 13 members of the AQP family and serves as a bidirectional water-selective transporter (Verkman et al., 2006; Verkman, 2011) and is heterogeneously expressed throughout the CNS with highest expression levels in the cerebellum and significantly lower protein levels in the hippocampus, diencephalons, and cortex (Hubbard et al., 2015). Additionally, the water channel protein is primarily associated with brain-fluid interfaces such as the BBB and ependymal-cerebrospinal fluid (CSF) barriers (Verkman, 2011) and is largely expressed by astrocytes predominantly at the perivascular end-feet in direct contact with blood vessels (Verkman et al., 2006; Nagelhus and Ottersen, 2013; Yao et al., 2015). Studies utilizing transgenic mice lacking AQP4 have provided a deeper understanding of this protein in various functions and diseases.

Using both *in vitro* and *in vivo* studies, researchers have identified an important role of AQP4 in astrocyte migration. A modified Boyden chamber was utilized for the transwell migration assay where astroglia migrated through a porous filter in response to a chemoattractant. Results from this assay revealed a slower migration of AQP4-null astrocyte as compared to wild-type (WT) astrocyte. Using a wound healing assay, confluent astroglial monolayers were injured by removing ∼1 mm of cells across the well. Glial scar formation by reactive astrocytes is crucial in wound healing. Results showed impaired wound healing and migration in AQP4-null astroglia. Additionally, the *in vivo* stab injury model also demonstrated a decrease in cell migration in AQP4-null mice (Saadoun et al., 2005).

What functional role does AQP4 play in cell migration? Early literature has shown localized swelling of the lamellipodia as a cause of cell movement (Oster and Perelson, 1987). Because AQP4 mediates the bidirectional transport of water, it is possible that migration of astrocytes is caused by increased water permeability leading to increased transmembrane water flux which ultimately moves the cell. Additionally, small extracellular osmotic gradients can also affect the speed of astrocyte movement in which astrocyte migration is accelerated toward hypo-osmolality (Saadoun et al., 2005).

Water homeostasis maintenance is critical in the CNS. Increases in brain water content can result in deleterious effects and thus understanding water regulation via AQP4 is of great significance. The role of AQP4 in brain edema has been heavily investigated in various studies using AQP4-null mice. There are two main types of cerebral edema: cytotoxic (cellular) edema and vasogenic edema. Cytotoxic edema results in an intracellular accumulation of water across an intact BBB whereas vasogenic edema results from fluid leaking across a compromised BBB (Verkman, 2005, 2008; Papadopoulos and Verkman, 2007). Studies of cytotoxic edema showed that AQP4-null mice were protected from cellular swelling and had improved neurological outcome (Manley et al., 2000, 2004; Papadopoulos and Verkman, 2005; Yao et al., 2015) while vasogenic edema worsens in mice lacking the water channel protein (Papadopoulos et al., 2004a,b). Hydrocephalus, a specialized form of vasogenic edema, results from obstruction of CSF drainage. Studies utilizing mice deficient in AQP4 showed accelerated progression of hydrocephalus, enlargement of ventricles, and increased intracranial pressure (Verkman et al., 2006).

While AQP4 can certainly play a significant role in edema formation, it is possible that AQP4 can also take part in edema elimination due to its function of bidirectional water transport. The traditional view of excess brain water elimination is believed to be through the bulk flow of fluid through the extracellular space (ECS) and the glial limitans, into the ventricles, and eventually into the blood through AQP4 located at the astrocytic endfeet (Verkman et al., 2006; Smith et al., 2015). This theory is supported by studies using models of brain edema and ischemia where electron microscopy findings demonstrate endfeet swelling of astrocytes suggesting AQP4-dependent mediated osmotic water uptake (Manley et al., 2000; Ito et al., 2011) and early induction of AQP4 reduced the development of edema formation (Hirt et al., 2009). The glymphatic hypothesis suggests that brain water elimination is by way of hydrostatic pressure and osmotic forces that drives water through AQP4 (Thrane et al., 2014, 2015). In addition to fluid elimination, the glymphatic hypothesis also provides a possible pathway for interstitial solute clearance from the brain that is also AQP4 dependent. For example, Iliff et al. (2012) demonstrated significant reduction of clearance of ^125^I-amyloid β_1-40_ in AQP4-null mice as compared to WT and AQP4-null amyloid-beta (Aβ) precursor protein/presenilin 1 transgenic mice had increased amyloid plaque deposition in the hippocampus and cortex as compared to WT animals (Xu et al., 2015). This suggests that interstitial soluble Aβ is removed via the gliovascular pathway in which clearance of interstitial fluid and solutes are driven by convective bulk interstitial fluid flow that is aided by AQP4-dependent water flux (Iliff et al., 2012). This hypothesis is further supported by mathematical modeling which considers the intracellular and extracellular water pathways between the arterial and venous paravascular space in astrocyte networks (Asgari et al., 2015).

AQP4 has also been associated with other neurological disorders such as epilepsy (Binder et al., 2012). Seizure phenotype of Aqp4-null mice was observed using the convulsant (GABA_A_ antagonist) pentylenetetrazole (PTZ). Interestingly, AQP4-null mice showed a longer latency to generalized seizure when given PTZ as compared to WT mice (Binder et al., 2004a). Furthermore, AQP4 has been shown to be colocalized with the inwardly rectifying potassium channel Kir4.1. The colocalization of these two proteins suggest that AQP4 contributes to the coupled influx of water and K^+^ after neuronal activity. Prolonged increases in [K^+^]_o_ in response to electrical stimulation were observed as well as an increased electrographic seizure threshold and electrographic seizure duration after stimulation. These findings suggests a deficit in extracellular K^+^ clearance in AQP4-null animals (Binder et al., 2006). Indeed, the brain is sensitive to changes in extracellular osmolarity as this would alter the cell volume and subsequently the ECS (Traynelis and Dingledine, 1989; Schwartzkroin et al., 1998). Changes in ECS volume significantly impacts the extracellular concentration of solutes. A multitude of solutes are released upon stimulation, including potassium, that are known to be cotransported with water (Haj-Yasein et al., 2012). Thus altered ECS has been correlated to brain tissue excitability (Binder et al., 2004b; Haj-Yasein et al., 2012). In a study using cortical fluorescence recovery after photobleaching method, ECS diffusion was reduced after glutamate and seizure-induced neuronal activity. Mice lacking AQP4 also exhibited faster ECS diffusion than WT suggesting an ECS expansion (Binder et al., 2004b).

Compelling evidence from these studies show that AQP4 not only control water and ion homeostasis but also plays a role in neural signal conduction that may potentially contribute to synaptic transmission that is regulated by water transport.

## AQP4 and Synaptic Plasticity

While there are mounting evidence that suggest a role of astrocytes in synaptic plasticity there are only a few studies that implicate a direct relationship of AQP4 in LTP and LTD. Here, we explore recent studies that present compelling data that support the hypothesis that AQP4 plays an important role in regulating synaptic plasticity in the hippocampus and amygdala. **Table 1** outlines the comparison of synaptic plasticity between WT and AQP4-null mice.

**Table 1:** AQP4 in synaptic plasticity.

Study	Genotype	Stimulation paradigm	Pathway	Results
Skucas et al., 2011	WT	TBS HFS LFS	SC-CA1 (*in vitro*) SC-CA1 (*in vitro*) SC-CA1 (*in vitro*)	LTP LTP LTD
	KO	TBS HFS LFS LFS + TrkB-Fc LFS + K252a	SC-CA1 (*in vitro*) SC-CA1 (*in vitro*) SC-CA1 (*in vitro*) SC-CA1 (*in vitro*) SC-CA1 (*in vitro*)	Reduced LTP with delayed LTD LTP LTD with delayed LTP LTD LTD
Fan et al., 2013	WT	TBS TBS	SC-CA1 (*in vivo*) PP-DG (*in vivo*)	LTP LTP
	KO	TBS TBS	SC-CA1 (*in vitro*) PP-DG (*in vivo*)	Reduced LTP Reduced LTP
Yang et al., 2013	WT	TBS TBS + Cef TBS + DHK	PP-DG (*in vivo*) PP-DG (*in vivo*) PP-DG (*in vivo*)	LTP LTP Reduced LTP
	KO	TBS TBS + Cef	PP-DG (*in vivo*) PP-DG (*in vivo*)	Reduced LTP LTP
Li et al., 2012	WT	HFS	Thalamo-LA	LTP
	KO	HFS HFS + Cef	Thalamo-LA Thalamo-LA	Reduced LTP LTP

*Comparisons of synaptic potentiation in wild-type (WT) and KO mice.*

### Basal Transmission in AQP4 WT and KO Mice

Using age-matched hippocampal slices Skucas et al. (2011) showed no differences in Schaffer collateral (SC) transmission between AQP4 WT and AQP4-null mice in extracellular recordings. There were no significant differences in field excitatory postsynaptic potentials (fEPSP) slope, amplitude, area, total duration, or half-duration. Additionally, there were no differences detected in the mean amplitude or latency to peak of the fiber volley, or the change in fiber volley amplitude with increasing fEPSP slope or amplitude. Furthermore, there were no significant differences in the paired-pulse facilitation (PPF), amplitude, area under the curve, or duration of both WT and AQP4-null slices. Whole-cell recordings from CA1 pyramidal cells also revealed no significant differences in frequency, amplitude, and cumulative probability of spontaneous postsynaptic currents and miniature postsynaptic currents (Skucas et al., 2011).

Fan et al. (2013) saw similar findings using hippocampal slices prepared from WT and AQP4-null mice. In their study, there were no differences in fEPSP slope between the two genotypes. Normal PPF was also observed in AQP4-null mice indicating that lack of the water channel protein did not cause a change in basal synaptic transmission (Fan et al., 2013).

Results from Yang et al. (2013) were parallel to findings from Fan et al. (2013). Basal synaptic transmission were not statistically different in AQP4 WT and AQP4-null hippocampal slices from the perforant path-dentate gyrus pathway (PP-DG) as there were no differences in fEPSP slope in both genotypes (Yang et al., 2013).

In a different study, Li et al. (2012) explored basal synaptic transmission in the thalamo-lateral amygdala (LA) pathway in slices taken from age and weight-matched littermates of WT and AQP4-null mice. As with findings reported above, fEPSP amplitude and PPF were not significantly different between WT and AQP4-null animals (Li et al., 2012).

These studies show that AQP4 deficiency does not cause a general defect in basal synaptic plasticity in various pathways in the brain due to undistinguishable differences in fEPSP and PPF between AQP4 WT and AQP4-null mice. Lack of AQP4, therefore, does not alter the probability of neurotransmitter release in the presynaptic neuron (Commins and O’Mara, 2000; Li et al., 2012; Scharfman and Binder, 2013). Thus, impairment in synaptic plasticity in AQP4-null mice might possibly be due to changes in the postsynaptic responses (Li et al., 2012). While these findings provide strong support that an absence of AQP4 does not affect basal synaptic transmission *in vitro*, data from *in vivo* studies could offer a more valuable assessment.

### AQP4 in Long Term Potentiation and Long Term Depression

To determine the role of AQP4 in synaptic plasticity Skucas et al. (2011) evaluated LTP and LTD from hippocampal slices of age-matched WT and AQP4-null mice using theta-burst stimulation (TBS) and high-frequency stimulation (HFS) in the SC synapse in CA1. Using the TBS-LTP paradigm, the authors noted no significant differences in post-tetanic potentiation (PTP) during the first 3 min, however, a reduction in LTP was observed in slices from AQP4-null mice at 60 min. Additionally, the incidence of LTP was reduced in AQP4-null mice. The authors also observed an unexpected delayed LTD in AQP4-null mice after TBS. To further evaluate this surprising observation, additional experiments were conducted in AQP4-null slices to assess fEPSP decay. The authors saw no significant decay in fEPSP slope and thus established the conclusion that TBS evoked LTD in KO mice (Skucas et al., 2011).

The authors further evaluated LTD using low-frequency stimulation (LFS) due to the unanticipated finding of LTD induction after TBS. Skucas et al. (2011) saw no significant differences in short-term depression but LTD was reduced in AQP4-null mice. Moreover, the incidence of LTD was also lower in these animals. Interestingly, the authors also saw a delayed LTP in AQP4-null mice using LFS. Finally, using HFS, Skucas et al. (2011) saw no significant differences in PTP, incidence of LTP, and LTP amplitude 60 min after HFS in both genotypes (Skucas et al., 2011).

Increasing evidence from studies of the neurotrophin brain-derived neurotrophic factor (BDNF) have implicated a role of BDNF-TrkB signaling in synaptic plasticity (Purcell and Carew, 2003; Minichiello, 2009; Park and Poo, 2013), however, the influence of AQP4 on activity-dependent BDNF-TrkB synaptic plasticity is only beginning to be elucidated. The first line of evidence, to our knowledge, that illustrates a direct impact of AQP4 on BDNF-dependent synaptic plasticity is demonstrated by Skucas et al. (2011). In their study, Skucas et al. (2011) asked if either scavenging BDNF or antagonizing TrkB would rescue LTD as it was shown that LFS resulted in a delayed LTP in AQP4-null mice. Using the BDNF scavenger TrkB-Fc the authors saw a rescue of LTD in AQP4-null mice. Furthermore, application of K252a, a Trk antagonist, also rescued LTD. Western blots for TrkB and immunoprecipitation/Western blots for P75NTR, a receptor for neurotrophins (Binder and Scharfman, 2004), were analyzed to determine if LTP and LTD in AQP4-null mice were influenced by levels of TrkB receptors. Western blots revealed no differences in full-length or truncated TrkB between WT and AQP4-null mice. However, immunoprecipitation/Western blots data showed reduced levels of p75NTR in AQP4-null animals (Skucas et al., 2011).

Fan et al. (2013) also observed impaired LTP in AQP4-null mice. LTP was induced using two different models; (1) in the SC-CA1 pathway *in vitro* and (2) in the PP-DG pathway *in vivo*. TBS in SC-CA1 resulted in a reduction of LTP in AQP4-null mice. There were also no differences in I/O curves between the two genotypes at different stimulation intensities. TBS-induced LTP in the PP-DG *in vivo* also resulted in impaired LTP in AQP4-null mice. An initial increase in population spike (PS) amplitude was observed immediately after TBS, however, the PS amplitude was significantly lower in AQP4-null mice as compared to WT. Furthermore, the potentiation of the PS amplitude remained significant in both WT and AQP4-null animals but there was significantly less LTP of PS amplitude in AQP4-null mice. These results suggest that AQP4 is involved in LTP induced by TBS in the DG *in vivo* (Fan et al., 2013).

Impaired LTP was also observed in TBS-induced LTP in the PP-DG *in vivo* by Yang et al. (2013). An initial increase of PS amplitude immediately after TBS also remained significant after 60 min in both genotypes. Similar to findings reported previously, LTP was greatly reduced in AQP4-null mice although both genotypes exhibited LTP after TBS. (Yang et al., 2013). It is well documented that glutamate plays a significant role in synaptic potentiation (Bear and Malenka, 1994; Perea et al., 2009; Achour and Pascual, 2010) and the high affinity glutamate transporter-1 (GLT-1) has been shown to be colocalized with AQP4 (Zeng et al., 2007). Furthermore, decreased expression levels of GLT-1 has been reported in AQP4-null mice which results in reduced glutamate uptake by astrocytes (Zeng et al., 2007; Papadopoulos and Verkman, 2013). Studies reported in this review thus far have shown impaired LTP in AQP4-null mice. Therefore, Yang et al. (2013) asked whether ceftriaxone (Cef), a β-lactam antibiotic that has been shown to up-regulate GLT-1 expression in astrocytes (Rothstein et al., 2005), can rescue AQP4 deficiency induced synaptic plasticity impairment. WT and AQP4-null mice underwent TBS in PP-DG *in vivo* after receiving daily injections of Cef for 7 days. PS amplitude was strongly increased in AQP4-null animals immediately and 60 min after TBS. Cef treatment did not further increase PS amplitude in WT animals. Furthermore, daily injections of dihydrokainate (DHK), a GLT-1 inhibitor, decreased PS amplitude in WT mice immediately and 60 min after TBS (Yang et al., 2013). These astounding results provide evidence that LTP impairment in AQP4-null mice can be rescued by Cef.

Li et al. (2012) also observed an impaired LTP in AQP4-null mice in the thalamo-LA pathway. After HFS, LTP was markedly reduced in AQP4-null slices as compared to WT slices that was not due to basal synaptic transmission as previously discussed. Similar to studies conducted by Yang et al. (2013), chronic Cef treatment reversed the impairment of LTP in AQP4-null mice (Li et al., 2012).

An outline describing LTP and LTD in both genotypes are reported in **Table 1**.

## Potential Mechanisms Underlying Impaired LTP in AQP4 KO Mice

### NMDAR-Dependent Synaptic Plasticity

It is well recognized that LTP and LTD induction is dependent on postsynaptic NMDA receptor (NMDAR) activation and the subsequent rise in intracellular calcium (Bear and Malenka, 1994; Lamprecht and LeDoux, 2004; Taniike et al., 2008; Paoletti et al., 2013). High increases in calcium activates various protein kinases which results in new AMPAR insertion into the postsynaptic membrane and ultimately leads to LTP while low increases of calcium activates protein phosphatases which dephosphorylates AMPAR and induces LTD (Bear and Malenka, 1994; Lamprecht and LeDoux, 2004; Taniike et al., 2008). It is possible that the lack of AQP4 could lead to NMDA dysregulation and consequently impaired LTP after TBS through reduced calcium entry. The attenuated LTP in response to TBS in hippocampal slices from AQP4-null mice could also account for NMDAR being less activated. This may result from impaired bicarbonate transport (Scharfman and Binder, 2013). During neuronal activity, increases in extracellular pH promotes NMDAR activation (Sinning and Hübner, 2013). Bicarbonate acts as a pH buffering system (Sinning and Hübner, 2013) and is regulated by the electrogenic Na^+^/HCO_3_^-^ cotransporter which drives water into astrocytes through AQP4 after taking up sodium and bicarbonate (Nagelhus et al., 2004). The absence of AQP4 could then affect pH homeostasis at the synapse during TBS.

Although postsynaptic activation of NMDAR is critical for LTP induction, excessive activation of these receptors may possibly contribute to the impairment of LTP seen in the AQP4-null mice. NMDARs have a higher affinity for glutamate compared to AMPARs (Patneau and Mayer, 1990). GLT-1 expression levels are reduced in APQ4-null mice (Zeng et al., 2007) and the downregulation of GLT-1 plays a significant role in synaptic plasticity as GLT-1 is responsible for the largest proportion of glutamate transport (Danbolt, 2001). Thus, large accumulation of glutamate, as a result of the reduced levels of GLT-1 in AQP4 deficient mice, results in intense activation of NMDAR (Danbolt, 2001). Strong NMDAR activation by excessive accumulation of glutamate was also observed in mice lacking GLT-1 (Katagiri et al., 2001). Additionally, increases in NMDAR-mediated currents was observed by Li et al. (2012) and Yang et al. (2013) in AQP4 deficient mice. Both studies measured the ratio of NMDAR to AMPAR mediated EPSC amplitudes to evaluate synaptic strengths. The authors found that NMDAR/AMPAR ratio was significantly larger in AQP4-null mice as compared to WT mice and that lack of AQP4 resulted in a selective increase in NDMAR-dependent EPSCs (Li et al., 2012; Yang et al., 2013). Moreover, low concentration of NMDAR antagonist reversed the impairment of LTP in AQP4-null mice (Li et al., 2012). These findings further support the hypothesis that excess glutamate in the synaptic cleft is due to the reduction of GLT-1 expression in AQP4-null mice which results in increased NMDAR-mediated currents in the hippocampus and the amygdala which may contribute to the impaired LTP in AQP4-null mice (Li et al., 2012; Yang et al., 2013). While the importance of synaptic NMDAR in synaptic plasticity is evident, the role of extrasynaptic NMDAR and glutamate is also critical in understanding synaptic potentiation. Elevated extracellular concentration of glutamate has been shown to strongly activate extrasynaptic NR2B-mediated NMDAR (Li et al., 2012). A correlation between the NR2B subunit and LTP has also been observed. Previous studies have shown that selective reduction in NR2B expression levels in the hippocampus disrupts LTP which consequently resulted in a decline in spatial learning behavior (Clayton et al., 2002). Thus, the impaired LTP seen in AQP4-null animals may also involve the NR2B subunit of the NMDAR (Li et al., 2012).

### Effect of Potassium Dysregulation in Synaptic Plasticity

Another essential role of astrocytes is maintaining potassium homeostasis. This is achieved by either net uptake of potassium or potassium spatial buffering (Macaulay and Zeuthen, 2012; Bedner and Steinhäuser, 2014; Cheung et al., 2015). The net uptake of potassium involves a variety of cotransporters that includes the Na^+^/K^+^ pumps as well as the Na^+^/K^+^/Cl^-^ cotransporters (Macaulay and Zeuthen, 2012; Bedner and Steinhäuser, 2014). In the spatial potassium buffering model (Orkand et al., 1966) extracellular potassium is taken up by Kir4.1 and redistributed to adjacent astrocytes via gap junctions (Bedner and Steinhäuser, 2014; Cheung et al., 2015). Therefore, potassium dysregulation could also possibly explain the impairment in synaptic potentiation seen in AQP4-null animals. Induction of LTP is related to neuronal excitability which is also sensitive to changes in extracellular potassium (Li et al., 2012). Previous studies have reported slowed K^+^ reuptake in AQP4-null mice (Binder et al., 2006) that may also be contributed by the Na^+^/K^+^ pumps (Strohschein et al., 2011). Reduced potassium reuptake would lead to an increase in extracellular potassium that would depolarize neurons and glial cells. High levels of [K^+^]_o_ would result in tonic depolarization of neurons which may improve LTP due to greater postsynaptic depolarization. However, tonic depolarization, by high levels of extracellular potassium, may also impair LTP by reducing the driving force of the fEPSP or inactivating sodium channels, which would decrease the postsynaptic firing during LTP induction (Scharfman and Binder, 2013). Certainly, LTP has been shown to be expunged by high [K^+^] (Harrison and Alger, 1993). Although AQP4 has been shown to colocalize with the inwardly rectifying potassium channel Kir4.1 (Nagelhus et al., 2004; Zhang and Verkman, 2008) the function of Kir4.1 does not seem to play a role of impaired potassium kinetics seen in AQP4-null mice (Zhang and Verkman, 2008). Furthermore, conditional Kir4.1-null slices exhibited defects during short-term plasticity indicating that Kir4.1 plays an essential role in potassium buffering during the early stages of LTP (Djukic et al., 2007).

### Contribution of GLT-1 and Ceftriaxone in Synaptic Potentiation

As previously stated, GLT-1 is responsible for reducing the spillover of glutamate at the excitatory synapses preventing excitotoxicity and ultimately controlling synaptic currents (Omrani et al., 2009). Previous studies have shown that AQP4 deletion resulted in a reduced expression of GLT-1 that led to an accumulation of extracellular glutamate (Zeng et al., 2007). Therefore, impaired LTP in AQP4-null mice may be explained by the reduction of glutamate uptake by glial cells. Other studies have also noted late LTP in GLT-1 deficient mice (Katagiri et al., 2001). Additionally, Rothstein et al. (2005) showed an impressive upregulation of GLT-1 by chronic treatment of Cef (Rothstein et al., 2005) which implicate a notable role of GLT-1 in synaptic plasticity. The effect of Cef treatment on LTP was seen in the studies conducted by Li et al. (2012) and Yang et al. (2013) where the authors noted that Cef treatment attenuated the deficits of LTP in the DG (Yang et al., 2013) and the LA (Li et al., 2012). Conversely, upregulation of GLT-1 by chronic treatment of Cef impaired LTD after LFS in the mossy fiber (MF)-CA3 pathway. Moreover, LTP was reduced after HFS in MF-CA3 but not in SC-CA1. Mechanisms underlying the induction of LTP in MF-CA3 is presynaptic, metabotropic glutamate receptors-dependent, and involves the release of glutamate, whereas LTP induction in SC-CA1 is primarily postsynaptic (Omrani et al., 2009). Additionally, LTP induction differs in different regions of the hippocampus. For example, LTP in SC-CA1 is NMDAR and glutamate dependent while LTP in MF-CA3 is NMDAR independent (Ota et al., 2013). Therefore, the reduced expression of GLT-1 in AQP4-null mice may affect LTP after TBS but have little affect after HFS (Omrani et al., 2009).

### BDNF-Dependent Synaptic Plasticity

Neurotrophins are a family of four structurally related proteins that include BDNF, nerve growth factor (NGF), neurotrophin-3 (NT-3) and neurotrophin-4/5 (NT-4/5; McAllister et al., 1999; Binder and Scharfman, 2004; Park and Poo, 2013). The neurotrophins bind to the lower-affinity p75NTR receptor and each bind to one or more of the high-affinity Trk family of receptor tyrosine kinases (McAllister et al., 1999; Binder and Scharfman, 2004; Minichiello, 2009; Park and Poo, 2013). NGF binds to TrkA, BDNF and NT-4 binds to TrkB, and NT-3 binds to TrkC (Binder and Scharfman, 2004; Park and Poo, 2013) and with low-affinity binding to TrkB (Binder and Scharfman, 2004). Additionally, there are two different classes of TrkB receptors; (1) full-length TrkB receptors that contains all the canonical motifs of tyrosine kinase receptors and (2) two alternatively spliced truncated receptors that lack the entire kinase catalytic region (Binder and Scharfman, 2004; Minichiello, 2009). Moreover, activation of the Trk receptors promote pro-survival signals while activation of p75NTR imparts anti-survival signals (Berk et al., 2015).

Multiple studies have recognized BDNF as a central player in regulating synaptic plasticity in the CNS. For example, mice targeted with a disruption of the BDNF gene displayed defects in the basal synaptic transmission and LTP at the SC-CA1 synapse which can be rescued by exogenous application of recombinant BDNF (Patterson et al., 1996). BDNF-TrkB activation is also regulated through PLCγ, PI3K, and MAPK pathways (Yoshii and Constantine-Paton, 2010). For instance, studies conducted by Ying et al. (2002) not only confirmed that BDNF is implicated in LTP but that activation of the MAPK pathway is required for the induction of LTP (Ying et al., 2002).

Again, it is interesting to note that impaired potentiation in AQP4-null mice was only observed in specific types of plasticity (TBS-LTP induced a delayed LTD and LFS induced a delayed LTP in AQP4-null mice; Skucas et al., 2011) and the roles of BDNF and TrkB could offer explanations to these differences. TBS-LTP attenuated LTP when TrkB functions are blocked, however, synaptic potentiation using HFS-LTP is relatively insensitive to perturbed TrkB functions (Kang et al., 1997). Thus, TBS-LTP appears to be dependent on BDNF (Scharfman and Binder, 2013). Furthermore, BDNF and TrkB also plays a significant role in early and late phase LTP. Blockade of TrkB functions results in impaired LTP by TBS as early as 15 min after LTP induction (Kang et al., 1997) and sustained release of BDNF, either presynaptically or postsynaptically, appears to induce and maintain late-LTP (Kang et al., 1997; Barco et al., 2005). Furthermore, while both BDNF and NT-4/5 bind to TrkB, there are stark functional differences between the two *in vivo* (Fan et al., 2000) and *in vitro* (Xie et al., 2000). Therefore, one cannot exclude the possibility that AQP4 may affect synaptic potentiation in NT-4/5 and NT-3 with TrkB signaling.

While there were no differences in TrkB receptor levels, the reduced levels of p75NTR could also explain the delayed LTD observed after TBS-LTP seen in KO mice. Studies have shown that binding of proBDNF, the precursor to mature BDNF, to p75NTR results in LTD (Pang et al., 2004; Woo et al., 2005; Yang et al., 2014). But because scavenging BDNF and antagonizing Trk rescued LTD after LFS, reduced levels of p75NTR is thus not necessary to cause the LTD defect seen in AQP4-null mice (Skucas et al., 2011). Furthermore, increased mRNA and protein levels of p75NTR was observed in hypoosmolar conditions (Ramos et al., 2007) which is comparable to an expanded ECS seen in AQP4-null mice (Binder et al., 2004b). Hypoosmolarity could ultimately dilute extracellular molecules (Scharfman and Binder, 2013) which presents as a plausible explanation for the changes in potentiation seen after TBS and LFS.

It is no surprise that cells undergo various changes when placed in hypoosmotic environments but how these conditions affect BDNF and synaptic plasticity remains unclear. Recent studies in retinal Müller cells may provide clues to explain for the deficits in synaptic plasticity seen in AQP4-null animals. For example, Berk et al. (2015) showed that BDNF inhibited osmotic swelling of retinal Müller cells. The somata of these cells did not alter much when they were challenged in a hypoosmotic condition. However, after blocking Kir channels with barium, Müller cell somata swelled significantly. Additionally, exogenous BDNF prevented barium-induced hypoosmotic swelling of the cells that was selective to TrkB (Berk et al., 2015). Furthermore, AQP4 has been shown to synergistically interact with the transient receptor potential isoform 4 (TPRV4) in Müller cells during glial swelling. Under hypotonic stress, water influx via AQP4 activates TPRV4 (which calcium enters through) and further promotes swelling (Jo et al., 2015). Moreover, calcium influx has also been show to modulate BDNF expression that is mediated by the activation of CREB (Shieh et al., 1998; Tao et al., 1998).

Although evidence of osmotic swelling inhibition by BDNF are still currently unknown, striking results from Berk et al. (2015) and Jo et al. (2015) could possibly link the relationship of BDNF and AQP4 in synaptic plasticity. Indeed, ionic flux coupled with water can alter the ECS and ultimately modulate neuronal activity (Andrew and MacVicar, 1994; Schwartzkroin et al., 1998; Dmitriev et al., 1999). The defect in LTD seen in AQP4-null animals (Skucas et al., 2011) could be a consequence of excess BDNF binding to TrkB during LFS to induce LTP but which was masked by the resulting LTD effect (Scharfman and Binder, 2013). The excess BDNF can also be explained through increases in calcium influx that promotes transcription of BDNF by CREB. Additionally, it is important to note that potassium mediated currents by Kir channels were blocked in Müller cells in the study conducted by Berk et al. (2015). Therefore, although the impaired potassium reuptake and altered water regulation was observed in AQP4-null mice could be of some agreement with the study, the enhanced ECS in AQP4-null mice seems to challenge the notion of osmotic swelling inhibition by BDNF. This could also be due to the difference in potassium kinetics in retinal Müller cells as compared to brain astrocytes. The mechanisms underlying excessive BDNF and swelling and its relationship to AQP4 remain to be resolved.

It is also well established that mature BDNF modulates LTP at the SC-CA1 synapse (Kang and Schuman, 1995; Jiang et al., 2003). Studies have demonstrated that release of mature BDNF leads to LTP (Kang and Schuman, 1995) and inhibits LTD (Jiang et al., 2003) and LFS suppresses the release of mature BDNF (Aicardi et al., 2004; Yang et al., 2013). Therefore, LTD induced by LFS was expected in WT mice, however, the delayed LTP observed in AQP4-null mice was unanticipated (Scharfman and Binder, 2013). Mature BDNF is known to be released by glial cells (Parpura and Zorec, 2010; Perea and Araque, 2010), hence, robust release of mature BDNF after LFS in AQP4-null mice might expound the observed LTP after LFS in AQP4-null slices (Scharfman and Binder, 2013). The data presented here suggests a tight regulation of pro and mature BDNF release for the competition of either LTP or LTD and that there may be a functional role of AQP4 in BDNF release in modulating synaptic potentiation.

**FIGURE 1:**
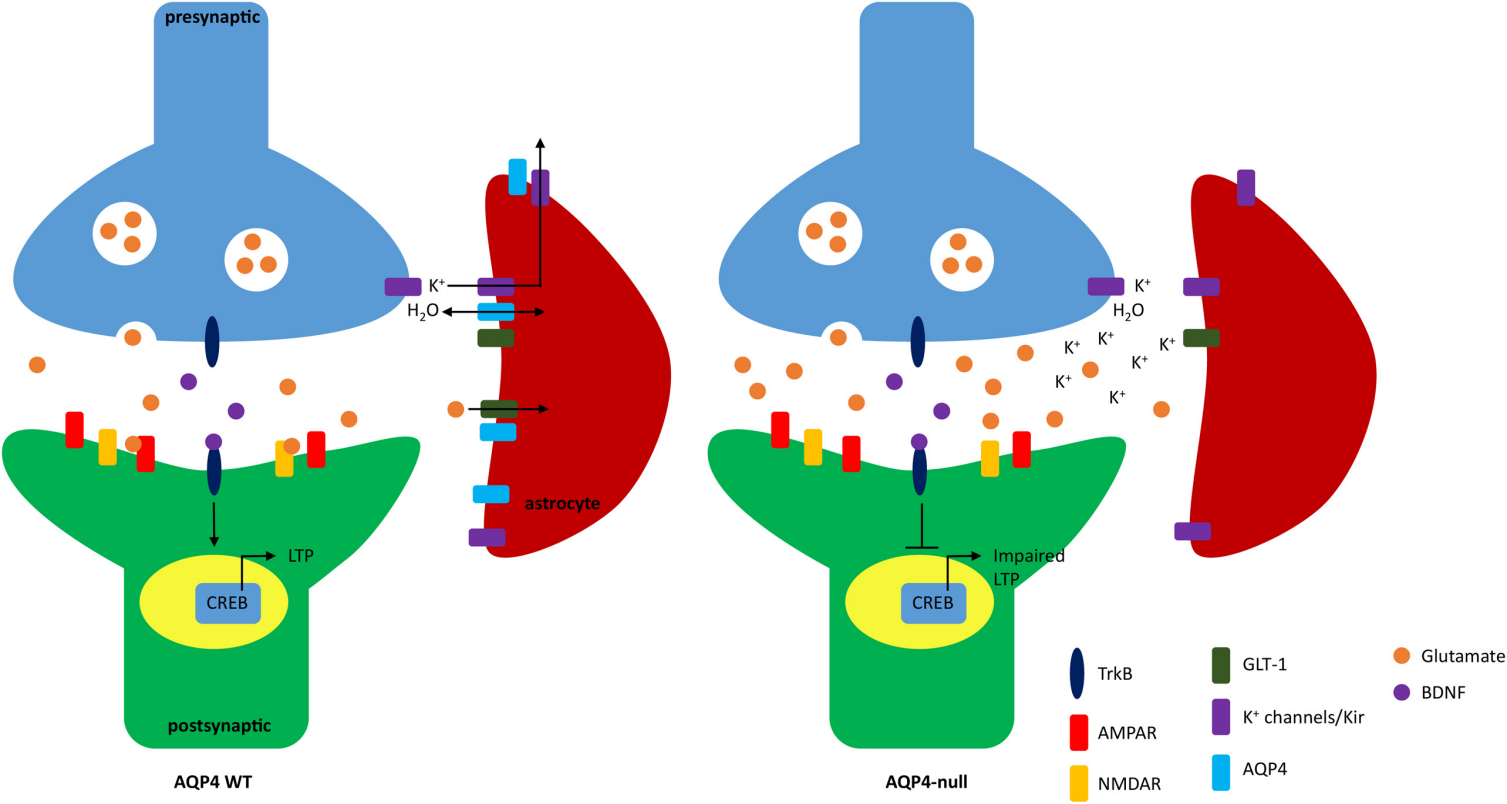
**Schematic of impaired long term potentiation (LTP) due to lack of AQP4.** In WT mice, AQP4 facilitates the bidirectional transport of water and is seen colocalized with GLT-1 and Kir4.1. Extracellular glutamate and potassium is taken up by GLT-1 and Kir4.1, respectively. Binding of glutamate to NMDA receptor (NMDAR) and AMPA receptors (AMPAR) increases calcium influx and promotes surface expression of AMPAR (not shown). The influx of calcium also modulates brain-derived neurotrophic factor (BDNF). Binding of BDNF to TrkB is regulated through three pathways: PLCγ, PI3K, and MAPK which activates the transcription factor CREB that further promotes gene expression to induce LTP. In AQP4-null animals potassium reuptake in astrocytes is impaired. NMDAR is also dysregulated which may result in loss of calcium influx and ultimately lack of BDNF-TrkB binding which inhibits CREB activation and downstream effects important to synaptic plasticity.

**Figure 1** depicts possible mechanisms of impaired LTP due to the absence of AQP4.

## AQP4 in Spatial Learning and Memory

Astrocyte dysfunction plays a major role in various neurological disorders that may affect synaptic plasticity. Since AQP4 has been shown to affect LTP and LTD, spatial learning and memory alterations in AQP4-null mice seems likely. To address this hypothesis, several studies have been conducted using different behavioral tasks to investigate the influence of AQP4 on cognitive functions. The Morris water maze (MWM) is a widely used method used to validate certain neurological conditions. The hippocampus has been suggested as the primary brain region for spatial memory acquisition and retrieval as well as memory storage and consolidation (D’Hooge and Deyn, 2001) and the hidden platform task of the WMW produces long lasting spatial memories (Kee et al., 2007). Fear conditioning is also a valuable technique in identifying neural circuits underlying synaptic plasticity in learning and memory particularly in the amygdala and hippocampus (Phillips and LeDoux, 1992; Maren, 2001; Pham et al., 2009). Finally, the hippocampal-dependent object placement (OP) task is useful in assessing cognition in spatial memory and discrimination and is also suitable for identifying memory alterations (Antunes and Biala, 2012). Behavior assessments from these tasks can help further elucidate the potential influence of AQP4 in synaptic plasticity and learning and memory.

### Morris Water Maze

Skucas et al. (2011) used the MWM to assess hippocampal-dependent behaviors. Mice were trained twice a day for 9 days with the platform in the same location. Animals were then subjected to a probe test 24 h after the acquisition phase to measure their retention. The authors found that both WT and AQP4-null mice had similar performances and that both groups reached a plateau at approximately day seven that were not statistically significant. Swimming speed, conducted during the probe test, was also not significantly different between genotypes (Skucas et al., 2011).

In a different study, Liu et al. (2012) used a model for Alzheimer’ disease (AD) to determine the effects of AQP4 in spatial learning and memory. Five month old female WT AQP4-null mice were first subjected to a bilateral ovariectomy (OVX) followed by daily injections of D-galactose (D-gal) 1 week after OVX for 8 weeks which was then proceeded by spatial memory testing using the MWM. High levels of D-gal, a reducing sugar in the body, results in the generation of a superoxide anion and oxygen-derived free radicals which ultimately produces brain oxidative stress-induced memory deficits. Control groups of WT and AQP4-null mice received sham operation and were treated with saline (Liu et al., 2012).

The training paradigm for the MWM, conducted by Liu et al. (2012), consisted of six consecutive days of training with four trails per day. The first 2 days of training were performed with a visible platform while the remaining 4 days utilized a hidden platform for testing. Escape latency, swim distance, swim speed, and swim patterns were analyzed. Genotype and treatment effects on motor ability and visual-spatial function was determined using the visible platform test. Swimming distance and escape latency only had an effect on training days and not on genotype or treatment. Additionally, there were no difference in swim speed in all groups during the first 2 days of training. This suggest that motor and/or visual deficits in adult mice were not due to OVX with D-gal treatment and AQP4 deficiency (Liu et al., 2012).

Spatial learning ability was then assessed in the hidden platform test. The authors found that escape latency and swim distance gradually decreased over the 4 days of training in all groups. WT and AQP4-null OVX plus D-gal treated groups required more time and distance to locate the hidden platform as compared to the vehicle treated control groups. Additionally, longer escape latency and swim distance was observed in AQP4 null-treated animals as compared to WT-treated animals, however, there was no difference between WT and AQP4-null control groups. Finally, swim speed was not affected by treatment or genotype demonstrating that swimming ability was unaffected and not related to spatial learning caused by OVX and D-gal treatment (Liu et al., 2012).

Swim patterns in the hidden platform tests was assessed to determine if delays in finding the hidden platform was associated with abnormal search pattern and deficits in OVX and D-gal-treated mice. WT and AQP4-null control groups swam within the inner portion of the pool with weaving or looping search patterns indicating that the control animals had learned the location of the hidden platform after training. For the OVX and D-gal-treated mice, particularly the AQP4-null group, the mice swam in random patterns suggesting the animals found the platform by chance. The random swimming pattern was observed throughout the last trial on day 6. Swim patterns reveals further information regarding memory deficits in each group of animals. Control mice tend to swim directly to the target quadrant to search for the hidden platform, whereas the OVX plus D-gal-treated mice swam in repetitive looping patterns to reach the target quadrant. These results indicate greater spatial learning and memory defects in OVX plus D-gal treated mice which were greater in AQP4-null mice. Finally, a probe test was conducted 1 h following the training trials on day 6 to assess whether a mouse had learned the location of the platform. WT and AQP4-null OVX plus D-gal treated mice spent a smaller percentage of time in the target quadrant and higher percentage of time in the adjacent quadrants as compare to the vehicle control mice. Additionally, WT-treated mice spent greater time in the target quadrant as compared to the AQP4 null-treated mice (Liu et al., 2012).

Fan et al. (2013) also employed MWM to investigate the correlation between AQP4 and memory consolidation between WT and AQP4-null mice. In this experiment mice were trained for four trails per day for five consecutive days. A probe trial was tested 24 h after the acquisition phase where the platform was removed and the mouse was allowed to swim for 60 s. One day after the first probe test, mice were trained for the reversal phase where the platform was removed and placed in the opposite quadrant used during the acquisition phase. Again, 24 h after the reversal phase, a second probe test was conducted. In another subset of mice a visible platform task was tested 24 h after the probe tests for two consecutive days where the platform was made visible using a black cubic landmark (Fan et al., 2013).

The authors noted a disruption in memory consolidation in the AQP4-null mice through analysis and measurement of the total time latency of the swim path during the acquisition and reversal phases. During the training period in the acquisition phase, WT mice showed improvement in their performance being able to reach the hidden platform faster over time while AQP4-null mice displayed a significantly shorter time of swim path compared to the WT only during the early stages of the acquisition phase. Similar to the acquisition phase results, AQP4-null mice had a shorter time of swim path in the early stages of the reversal phase as compared to WT. However, after comparing each trial, the authors noted that the AQP4-null mice seemed to forget the escape position more easily than the WT mice even though both genotypes were able to find the hidden platform. The probe tests also revealed impaired spatial retention in mice deficient in AQP4. The latency to cross the hidden escape platform was longer in AQP4-null mice as compared to the WT but the numbers of crosses were less in the AQP4-null mice. Furthermore, AQP4-null mice did not show preference toward the trained quadrants as the time spent in the quadrants were less than the WT. Finally, there was no difference in performance during the visible platform task in both genotypes which suggests that the AQP4-null mice presented learning deficits as compared to the WT (Fan et al., 2013).

Zhang et al. (2013) continued investigations from Fan et al. (2013) testing the hypothesis that AQP4 modulates the aversive motivation in MWM. The authors used two MWM testing paradigms; (1) hidden platform training (acquisition training with reward) and (2) non-platform training (swimming only without reward). In both MWM training protocols, WT and AQP4-null mice were trained for four trials a day for 9 days. A probe test was conducted on day 10. On days 11 and 12, a cued platform training was performed where the platform was made visible and moved to the opposite quadrant from the training quadrant. A different subset of WT and AQP4-null mice were used for the non-platform training. The latency to the hidden platform, distance moved, and the mean swimming velocity was measured and analyzed. The authors considered that reaching the hidden platform to be rewarding and thus the motivation to reach the reward would be measured by the mean swimming velocity (Zhang et al., 2013).

Similar to findings from Fan et al. (2013) and Zhang et al. (2013) also reported a shorter latency to the hidden platform in AQP4-null mice during the first day of training and a longer escape latency during the last training days. Although both genotypes showed progressive improvement in escape latency, there were no significant differences between WT and AQP4-null mice. Additionally, both genotypes displayed an increase in shorter pathways during the training phase; AQP4-null mice, surprisingly, traveled a significantly shorter distance than the WT mice. No differences in distance traveled to the platform was observed during the later training days in both genotypes. Swimming velocity was significantly different in both genotypes during the latter 7 days of the training period. WT mice had increased swimming velocity over the course of the acquisition phase while AQP4-null mice displayed a decrease in swimming velocity. The increased escape latency in AQP4 could be attributed to the decreased swimming velocity. These findings suggests that impairment of spatial learning and reduction of motivation could be associated with a lack of AQP4 in mice (Zhang et al., 2013).

In the non-platform MWM task, AQP4-null mice exhibited a significant decrease in their mean swimming distance and velocity. There were no differences in swimming distance or velocity between the WT and the AQP4-null during the first day of training. While both genotypes demonstrated the same length in swimming during the four-trial sessions, the AQP4-null mice showed a gradual reduction in travel length during the later training periods (Zhang et al., 2013).

To further analyze spatial learning, Zhang et al. (2013) used the probe test to measure memory retention in WT and AQP4-null from both MWM training. After the acquisition phase in the hidden platform task (reward), the probe test revealed that WT mice preferred the target quadrant. WT mice displayed more time and more distance traveled in the target quadrant. Additionally, WT mice showed more numbers of platform crossed as compared to AQP4-null mice. In the non-platform task (no reward), both WT and AQP4-null mice showed no preference to any of the four quadrants (Zhang et al., 2013).

Motivation of WT and AQP4-null animals during the probe test was then explored through analysis of swimming distance and velocity. There were no significant differences in swimming distance and velocity in WT mice. AQP4-null mice in the non-platform (swimming only without reward) training displayed a significantly shorter swimming distance and velocity as compared to AQP4-null mice that were trained with the hidden platform (reward). Furthermore, while AQP4 KO mice in the hidden platform task showed a reduction in swimming velocity, there was no significant differences in swimming velocity as compared to WT mice in the same hidden platform training. A possible explanation could be due to the fact that WT mice were decreasing their swimming speed while AQP4-null mice were increasing their swimming speed on their last training day prior to the probe test (Zhang et al., 2013).

Finally, the authors subjected the animals to two trials of the cued platform task where a visible platform was moved to the opposite quadrant from the training quadrant. During the first trial, escape latency, swimming distance, and swimming velocity did not differ between WT and AQP4-null mice that had undergone acquisition training (hidden platform with reward). In contrast, mice that were subjected to swimming only training (no reward) had a greater escape latency compared to animals that had acquisition training. Furthermore, in the group trained without reward, WT mice displayed a greater distance traveled compared to AQP4-null mice, however, by the second trial, WT mice had shorter distance travel path to the platform. WT mice trained without reward also showed an increase in swimming velocity compared to AQP4-null mice trained without reward. Escape latency and swimming velocity did not differ between genotypes in both training paradigms on the second trial. These findings suggest that acquisition training can improve the animals’ ability to reach the cued platform (Zhang et al., 2013). A summary of the MWM findings are listed in **Table 2**.

**Table 2:** Summary of findings from Morris water maze.

Study	Findings
Skucas et al., 2011	1. No significant differences between genotypes
Liu et al., 2012	1. No significant differences between genotypes during the visible platform test. 2. No significant differences between genotypes and treatment during the hidden platform test. 3. KO-treated animals had longer escape latency and swim distance. 4. KO-treated mice had higher random swim pattern. 5. KO-treated mice spent less time in target quadrant.
Fan et al., 2013	1. KO mice had shorter swim path in earlier stages of training. 2. KO mice had longer escape latency. 3. KO mice spent less time in target quadrant. 4. No significant differences in performance between genotypes during the visible platform task.
Zhang et al., 2013	1. No significant differences in escape latency between genotypes (hidden platform). 2. KO mice traveled shorter distances (hidden platform). 3. KO mice had significantly decreased swimming distance and velocity (hidden platform). 4. KO mice displayed gradual reduction in travel length (non-platform). 5. No significant differences between genotypes for quadrant preference (non-platform). 6. No significant differences between genotypes in escape latency, swimming, distance, and swimming velocity during cued platform task (hidden platform). 7. Wild-type mice had greater distance traveled compared to KO mice on first day of cued platform task (non-platform). 8. WT mice had increased swimming velocity compared to KO mice on first day of cued platform task (non-platform). 9. No significant differences in escape latency and swimming velocity between genotypes on second day of cued platform task (hidden platform and non-platform).

*Comparison of findings from the Morris water maze in WT and KO mice.*

### Contextual Fear Conditioning

Skucas et al. (2011) also performed contextual fear conditioning (CFC) on the same group of WT and AQP4-null mice 2–3 weeks after MWM testing (see above; Skucas et al., 2011). In this CFC protocol, mice were placed into the conditioning chamber and received three 2 s, 0.75 mA scrambled footshocks 2.5, 3.5, and 4.5 min after placement into the chamber. During the retention test (24 h after training), the animals received one 5 min exposure to the chamber without footshocks (Pham et al., 2009). CFC testing revealed that AQP4-null mice were more immobile than the WT during the conditioning phase, however, the levels of immobility between the two genotypes were not statistically significant (Skucas et al., 2011) suggesting that AQP4-null mice had normal long-term memory for contextual fear (Scharfman and Binder, 2013).

Using a different CFC method, Yang et al. (2013) observed contrasting results as compared to Skucas et al. (2011). In their study, mice were trained and tested in the conditioning chambers for two consecutive days. During the training period, the animals were exposed to the conditioning chamber for 3 min followed by a 2 s, 1.0 mA constant current foot shock. Memory test was performed 24 h after the training period by re-exposing the mice to the conditioning chamber for 3 min with the absence of the foot shock. Here, AQP4-null mice showed pronounced decreased freezing behavior as compared to WT mice. The results from this study suggests that AQP4 deletion impairs associate fear memory formation. Additionally, immobility in AQP4-null mice treated with Cef daily for a week had increased significantly. WT mice treated with Cef increased freezing behavior whereas WT mice treated with DHK decreased immobility. (Yang et al., 2013). These findings indicates that Cef may have therapeutic benefits in rescuing hippocampal-dependent memory deficits through increasing GLT-1 expression.

Using a light fear conditioning protocol, Li et al. (2012) determined if amygdala-dependent learning behavior is altered in AQP4-null mice. During the conditioning period, mice were allowed to explore the conditioning chambers for 3 min followed by a light conditioned stimulus (CS) which was produced by an 8 W white light bulb that was presented for 30 s and co-terminated with a single electric foot shock (0.7 mA, 1 s). The light-cued fear memory was tested 2 and 24 h after conditioning and freezing behavior was monitored for 3 min with the presentation of the light. Prior to CS, both WT and AQP4-null mice did not exhibit any significant differences in baseline behavior. During training, both genotypes displayed increased freezing behavior, however, differences in immobility were not statistically significant between WT and AQP4-null mice suggesting that normal acquisition of cued fear memory was not altered in AQP4-null mice. Freezing behavior was also unaltered in WT and AQP4-null mice 2 h after training, however, AQP4-null mice displayed reduced immobility 24 h after conditioning indicating that consolidation of associate fear memory is impaired in AQP4-null mice (Li et al., 2012).

To corroborate the findings that the freezing behavior was specific to fear-associated learning, the authors also performed an open field test, elevated plus maze, and a nociception test. A 10 min analysis in locomotive behavior during the open field test demonstrated no differences in locomotive activity between WT and AQP4-null mice. The results suggests that hyperlocomotive activity was not a factor in the decreased freezing behavior seen in AQP4-null mice. Elevated plus maze also resulted in no significant differences between WT and AQP4-null mice indicating unaltered level of innate fear and anxiety in AQP4-null mice. Finally, pain thresholds as assessed through vocalization and jump responses to increased intensity of electric shocks were not statistically significant between the two genotypes indicating that pain sensitivity does not influence fear memory impairment in AQP4-null mice (Li et al., 2012).

### Object Placement

The OP test was also performed in WT and AQP4-null mice by Skucas et al. (2011) using standard methods with a 1 or 24 h interval between the two trials (Scharfman et al., 2007). During the first trial, WT and AQP4-null mice spent the same percent of time exploring the objects when first presented. During the second trial (1 h interval), WT mice spent more time exploring the moved object while AQP4-null mice did not show preference toward the moved object. During the 24 h interval, WT and AQP4-null mice spent similar time exploring the objects. However, during the second trial, WT mice again spent more time exploring the moved object (Skucas et al., 2011). These results indicate that WT mice can differentiate between objects in a familiar and new location and that there is a defect in object placement memory in the AQP4-null mice (Scharfman and Binder, 2013).

## AQP4 Regulation in Cognitive Functions

Behavioral tasks findings between WT and AQP4-null mice varied between each study. Groups that utilized the MWM all saw impairment in spatial memory except for Skucas et al. (2011). Additionally, Skucas et al. (2011) also observed no significant differences between WT and AQP4-null mice during CFC while Li et al. (2012) and Yang et al. (2013) reported evident differences in immobility between WT and AQP4-null mice. Finally, OP test revealed defects in object placement memory as reported by Skucas et al. (2011).

Impaired cognitive function observed by Liu et al. (2012) in both WT and AQP4-null OVX plus D-gal treated animals can be correlated to decreased expression of the presynaptic vesicle protein synaptophysin (SYP) and the postsynaptic density protein-95 (PSD-95; Liu et al., 2012). These proteins are known to be altered in the hippocampus and cause memory deficits during the progression of AD (Sze et al., 1997; Xu et al., 2015). Another study of AD proposed a pathway including PSD-95, BDNF, and NMDAR. In this model, NMDAR stimulation recruits TrkB to the synapse and initiates BDNF signaling through the PI3K pathway to transport new PSD-95 to the synapse where it acts as a scaffold for BDNF receptors (Yoshii and Constantine-Paton, 2010). Decreased expression of SYP and PSD-95 were reported in both WT and AQP4-null OVX-treated mice, however, the reduced expression of these two proteins were more pronounced in AQP4 deficient animals which is consistent with the decline in spatial learning and memory (Liu et al., 2012). The deficits in cognitive function of these animals can be attributed to the lack of new PSD-95 to further strengthen the responsiveness of the synapse to BDNF to promote LTP.

Additionally, the cholinergic system has been linked to cognitive deficits in AD (McKinney, 2005; Schliebs and Arendt, 2006) and is also vulnerable to oxidative damage (McKinney, 2005). Interestingly, cholinergic neurons have also been associated with endogenous levels of estrogen (Gibbs, 1998) which has been correlated to BDNF and memory functions (Bekinschtein et al., 2007; Francis et al., 2012; Luine and Frankfurt, 2013). The OVX treatment performed by Liu et al. (2012) could reduce levels of BDNF which is mediated by activation of CREB (Shieh et al., 1998; Tao et al., 1998) and sublethal accumulation of Aβ has been shown to suppress activation of CREB (Tong et al., 2004). Additionally, the authors have observed a significant reduction of cholinergic neurons as well as increased brain oxidative stress and reduced antioxidative capabilities in AQP4-null-treated mice as compared to WT-treated mice and control animals. Therefore, an increase in Aβ in the hippocampus (a hallmark of AD) and brain oxidative stress with a decrease in SYP, PSD-95, and cholinergic neurons can be attributed to a lack of AQP4 in regulating astrocytic functions (Liu et al., 2012).

Studies performed by Fan et al. (2013) demonstrated significant memory consolidation impairment in AQP4-null mice as compared to WT mice. In particular, AQP4 deficient mice had an obvious dissociation between memory acquisition and spatial retention as assessed by the MWM. The authors stated that these findings can be attributed to the impaired hippocampal TBS-LTP *in vivo* (PP-DG) and *in vitro* (SC-CA1) and suggest that AQP4 may act downstream of glutamate receptors to regulate LTP memory formation and consolidation (Fan et al., 2013). The DG plays a fundamental role in memory storage and acquisition and is the site of neurogenesis (Kee et al., 2007; Jessberger et al., 2009; Fan et al., 2013). Studies have shown that as new neurons mature they are incorporated into the spatial memory circuits (Kee et al., 2007) and inhibiting neurogenesis in the DG results in impaired spatial and object recognition (Jessberger et al., 2009). Furthermore, AQP4 is expressed in adult neural stem cells (ANSCs) and has been shown to be involved in neurogenesis (Zheng et al., 2010) and participating in various vital roles such as neuronal migration (Saadoun et al., 2005; Zheng et al., 2010). Even though the authors did not observe alterations in the number of neural stem cells after MWM in either genotype, they deduced that the absence of AQP4 could possibly block the recruitment of new neurons to the spatial memory circuits that ultimately contributes to memory processing in the DG. The results indicate that ANSCs were not recruited into the DG memory circuit and that neuronal proliferation was inhibited (Fan et al., 2013). These findings can be validated by the previously mentioned roles of AQP4. The impaired memory consolidation observed in the AQP4-null mice may be a consequence of the lack of AQP4 in promoting cell migration and proliferation which would eventually inhibit the recruitment of ANSCs into the spatial memory network in the DG to stabilize memory trace (Fan et al., 2013).

Subsequent studies by Zhang et al. (2013) confirmed the dissociation between acquisition and spatial retention in APQ4-null mice that was observed by Fan et al. (2013). The authors noted significant reduction in swimming velocity in the AQP4-null mice as compared to the WT mice which they attributed to be a deficit in aversive motivation. During the first day of training in the hidden platform test the AQP4-null mice were more capable in finding the platform, however, their performance levels on escape latency was not comparable to the WT animals which resulted in reduced swimming velocity. Additionally, AQP4-null mice also showed slower swimming velocity during the swimming only task. The authors suggested that the AQP4-null mice “gave up” during a difficult task implying a lack of motivation (Zhang et al., 2013). Dopamine has been highly regarded in learning and motivation (Dayan and Balleine, 2002; Wise, 2004) and the regulation of dopamine has been correlated to AQP4 (Fan et al., 2005, 2008). Previous studies using AQP4-null mice have reported increased basal extracellular levels of dopamine (Fan et al., 2005; Ding et al., 2007), however, the correlation between increased levels of dopamine and motivation remains elusive (Cagniard et al., 2006; Treadway et al., 2012).

The hippocampus and the LA have both been implicated in the neural circuit of fear memory (Phillips and LeDoux, 1992). Although Skucas et al. (2011) observed an increase in immobility in both WT and AQP4-null mice during the testing trial of the CFC, the differences between the two genotypes were not statistically significant. On the other hand, Yang et al. (2013) observed contrasting findings. In their study, AQP4-null mice had a significant reduction in freezing behavior as compared to WT mice, however, this was rescued by chronic treatment of the GLT-1 activator Cef (Yang et al., 2013). The inconsistency in findings between the two studies could be due to different testing protocols. In another study, Li et al. (2012) also observed an increased in immobility in WT and AQP4-null mice, however, the AQP4-null mice had a significant reduction in freezing behavior 24 h after training as compared to WT mice. Similarly, Cef treatment rescued the impairment in mice lacking AQP4 (Li et al., 2012). Findings from Li et al. (2012) further solidified the hypothesis that GLT-1 is a key player in the mechanisms underlying AQP4 regulated synaptic plasticity and memory formation. Finally, the differences in findings between these studies are not entirely surprising as the discrepancies lies in the specific fear conditioning protocol. The hippocampus is known to be dependent on contextual fear memories while the LA is dependent on cued fear memories. Therefore, the mechanisms for the retrieval of the fear memory for either the hippocampus or the LA are not identical (Phillips and LeDoux, 1992; Hall et al., 2001; Li et al., 2012).

Memory impairment was only observed in the AQP4-null mice during the OP task and not the MWM or CFC in the study conducted by (Skucas et al., 2011). Associating the deficit to the short time interval during training and testing seems unlikely since the CFC was also tested during a 24 h interval. This may also be correlated to the early phase of LTP after TBS [60 min after induction (Scharfman and Binder, 2013)]. Nonetheless, the impairment observed during the OP task may be related to the different neural circuitry involved in this specific task as compared to MWM and CFC. Interestingly, the mechanisms underlying memory performance in OP task has been linked to BDNF (Bekinschtein et al., 2007; Francis et al., 2012; Luine and Frankfurt, 2013).

The studies presented here thus far have provided great insight to the role of AQP4 in learning and memory in hippocampal and amygdaloid-dependent tasks. While there are discrepancies between studies one must recognize that behavior performances from animals can be influenced by various factors such as differences in animal (strain, sex, age) and experiment protocols (differences in training). For example, consideration of mouse strain when interpreting data in specific behavioral tasks is imperative as different strains tend to exhibit marked differences in performances (Adams et al., 2002; Patil et al., 2008, 2009). Furthermore, defects in LTP, LTD, and behavioral tasks are not always entirely correlated to each other (Jun et al., 1998; von Engelhardt et al., 2008; Leiva et al., 2009). Despite the varied findings all groups have concluded that the lack of AQP4 results in cognitive deficits and these data have shed some light into the possible role of AQP4 in regulating learning and memory.

## Conclusion

AQP4 is the major water channel in the CNS and it is now established that AQP4 possesses greater functions beyond regulating water homeostasis in the brain. It is clear that the absence of AQP4 plays a unique role in synaptic plasticity and learning and memory although the exact mechanisms remain unclear. These emerging studies provides a glimpse into the potential role of AQP4 in LTP, LTD, and cognitive functions that was once elusive.

In reviewing the findings from these studies, it is evident that synaptic plasticity and learning and memory seems to be, in part, regulated by AQP4. For example, the fundamental basis of LTP induction requires NMDAR activation, however, mechanisms underlying the impairment of LTP in AQP4-null mice seems to be an indirect consequence from the lack of AQP4. In particular, the down regulation of GLT-1 and the subsequent elevation of glutamate in the ECS results in the strong activation of NMDAR which seems to inhibit LTP in KO mice. Furthermore, defects in potassium homeostasis plays a role in different stages of LTP. Finally, the neurotrophin BDNF is also observed as a key player in modulating LTP which may be associated with cellular swelling. Moreover, impairment of LTP observed in AQP4-null mice was followed by memory decline as assessed by MWM, fear conditioning, and object placement tasks, reiterating the potential role of AQP4 in cognition. While convincing studies reveal a critical role of AQP4 in synaptic plasticity and learning and memory, the mechanisms underlying the cellular and behavioral changes in mice lacking this astrocyte water channel is still unknown.

The interest of AQP4 in synaptic plasticity and cognition is also critical from a public health standpoint. AQP4 has been implicated in various neurological disorders such as epilepsy, cerebral edema, and Alzheimer’s disease. The effects of AQP4 in learning and memory are only beginning to be elucidated, therefore, ongoing research efforts is of great importance to the clinical field as there may be potential therapeutic benefits that may modulate this protein. And while there are certainly compelling evidence from these studies future investigations are required to further understand the precise role of AQP4 in synaptic plasticity and learning and memory.

## Author Contributions

JS performed a complete literature review and drafted the manuscript. DB conceived of the manuscript and provided feedback on its content.

## Conflict of Interest Statement

The authors declare that the research was conducted in the absence of any commercial or financial relationships that could be construed as a potential conflict of interest.
